# Altitude-mediated soil microbe-nutrient dynamics shape medicinal properties of *Angelica sinensis*

**DOI:** 10.3389/fpls.2025.1703258

**Published:** 2026-01-28

**Authors:** Xiao-Fang Gong, Wasim Khan, Ling Yang, Yu-Kun Chen, Juan Chen, Ling Zhang, Yong Zhang, Ying Zhu, Zhi-Ye Wang, Bing-Lin Zhang, Lin-Gui Xue

**Affiliations:** 1 School of Biological and Pharmaceutical Engineering, Lanzhou Jiaotong University, Lanzhou, China; 2 Institute of Biology, Gansu Academy of Sciences, Key Laboratory of Microbial Resources Exploitation and Utilization in Gansu Province, Lanzhou, China; 3 State Key Laboratory of Herbage Improvement and Grassland Agro-Ecosystems, College of Ecology, Lanzhou University, Lanzhou, China; 4 College of Life Sciences and Engineering, Hexi University, Zhangye, China; 5 Yulong Snow Station of Cryosphere and Sustainable Development, State Key Laboratory of Cryospheric Science and Frozen Soil Engineering, Northwest Institute of Eco-Environment and Resources, Chinese Academy of Sciences, Lanzhou, China

**Keywords:** *Angelica sinensis*, elevation, high throughput sequencing, microbial community, microbial diversity, pharmacodynamic component, soil factor

## Abstract

**Background:**

Rhizosphere microorganisms play a critical role in plant growth and medicinal quality, yet their altitudinal patterns and interactions with soil nutrients and bioactive compounds in *Angelica sinensis* (*A. sinensis*) remain poorly understood.

**Methods:**

Using Illumina MiSeq sequencing, we analyzed bacterial, fungal, arbuscular mycorrhizal (AM) fungal, and archaeal diversity across an altitudinal gradient, alongside soil physicochemical characteristics and bioactive components.

**Results:**

As cultivation elevation increased, bacterial and fungal diversity initially increased significantly and then stabilized (*p* < 0.05). In contrast, AM fungal and archaeal communities remained relatively stable. Bacterial communities varied significantly across altitudes (stress < 0.1, *p* = 0.001), as did soil nutrients and enzyme activities (*p* < 0.05). Bioactive components, except for ferulic acid, varied significantly with altitude. Redundancy analysis (RDA) confirmed that altitude and soil factors are key drivers of microbial community assembly. Mantel tests and structural equation modeling (SEM) demonstrated significant correlations between soil properties, microbial diversity, and medicinal properties of *A. sinensis* (*p* < 0.05).

**Conclusion:**

The mid-to high elevation zone (2520–2717 m) was identified as optimal for both yield and bioactive compound accumulation. These findings deepen the understanding of how microbes adapt to different altitudes in medicinal plants and offer a framework for precise cultivation of *A. sinensis*, thereby supporting the high-altitude symbiosis theory.

## Introduction

*Angelica sinensis* (Oliv.) Diels (Umbelliferae), a perennial herb with a 2000-year history of medicinal use, holds a unique status in traditional Chinese medicine (TCM) as demonstrated by its inclusion in the classical prescriptions summarized as “ten formulas and nine returns (Chinese Pharmacopoeia Commission, 2020). Its dried roots contain distinctive phthalides such as ligustilide and levistolide, as well as phenolic acids like coniferyl ferulate, which together exhibit multiple therapeutic effects, including regulation of blood formation, anti-inflammatory activity, and neuroprotection (Hook, 2014). *A. sinensis* prefers cold and humid environments (Xu et al., 2021a). Min County, located in Gansu Province, is the primary production area for *A. sinensis* in China. *A. sinensis* cultivated in Min County, known as “Min Gui”, is recognized as the gold standard for quality. This is largely due to its specialized high-altitude cultivation at elevations of 2200–2800 m, where cold and humid conditions promote superior accumulation of bioactive compounds (Xie et al., 2019; Liu et al., 2021a; Wang et al., 2012). However, current cultivation practices lack precision due to limited understanding of how elevation-driven microbiome and soil interactions affect medicinal quality. This study addresses this critical knowledge gap.

Rhizosphere microorganisms are recognized as the “second genome” of plants and are estimated to regulate nearly 80% of secondary metabolite biosynthesis (Wang et al., 2024a), play a crucial role in the growth and development of plants (Li et al., 2024a; Tong et al., 2021), and their community structure and metabolic activities have important effects on plant growth and secondary metabolite biosynthesis (Zhang et al., 2023; Li et al., 2022). Jiang et al. (2021) and Zhu et al. (2021) found that the rhizosphere soil bacterial community of *A. sinensis* has a significant influence on its growth, medicinal ingredients, and their types, under different cultivation conditions. The community composition and metabolic activity of rhizosphere microorganisms are affected by plant physiological status, geography, and climate (Feng et al., 2020). In northwest China, elevation critically impacts plant growth and medicinal quality by modulating temperature, precipitation, and light regimes (Zhang et al., 2024). While elevation-driven shifts in soil microbial communities exhibit predictable zonation patterns (Zhang et al., 2022a; Wei et al., 2024; Hou et al., 2024; Jiang et al., 2018), research on *A. sinensis* has largely focused on climatic and edaphic impacts (Liu et al., 2021a; Xi et al., 2021), and the effects of different elevations on the diversity and community structure of microbes in the *A. sinensis* rhizosphere soil have not been reported to date.

In this study, we systematically investigate interactions among an altitudinal gradient (2200–2800 m), rhizosphere microecosystems, and the biosynthesis of bioactive compounds in *A. sinensis*, addressing a significant knowledge gap in the research on alpine medicinal plants. Unlike previous elevation studies that focused primarily on individual environmental factors, we employ an integrated microbial community-soil properties-phytochemical approach to: (1) decipher how elevational zonation restructures the diversity and assembly of bacterial, total fungal, and archaeal communities via the distribution of soil nutrients and enzyme activities; (2) quantify the effects on the biosynthesis of key pharmacodynamic compounds; and (3) establish a predictive model linking soil physicochemical parameters and microbial community dynamics to the concentrations of the principal medicinal constituents of *A. sinensis* through SEM.

## Materials and methods

### Study site and sample collection

The experimental site was located in the *A. sinensis* cultivation area of Gansu Province, China. It is situated in a transitional zone from a temperate semi-humid climate to an alpine humid climate, characterized by cold and damp conditions, with an average annual temperature of 5.5°C, total annual precipitation of 635 mm, and average relative humidity of 68%. The soil type is chernozem, characterized by fine clay and low sand content. Six experimental sites at different altitudes ranging from 2210 to 2887 meters (m), including 2210 m (H1, N 34° 13′ 35″, E 104° 14′ 8″), 2425 m (H2, N 37° 1′ 1″, E 103° 7′ 38″), 2520 m (H3, N 34° 17′ 25″, E 104° 2′ 21″), 2615 m (H4, N 34° 31′ 22″, E 104° 28′ 50″), 2717 m (H5, N 34° 38′ 1″, E 103° 28′ 52″), and 2887 m (H6, N 34° 42′ 3″, E 104° 6′ 4″), were selected to transplant *A. sinensis* seedlings (variety of Mingui No. 1) with uniform transplanting density and under the same manual weeding regime. Samples were collected from H1 to H6 at the medicinal period (28 September 2024) of *A. sinensis*, using the five-point “S”-shaped sampling method, and five plants were combined to form one sample, and six samples were collected from each elevation site (H1–H6), resulting in a total of 30 samples. During sampling, *A. sinensis* plants with rhizosphere soil were placed in sterile, labeled, self-sealing bags and brought back to the laboratory in an icebox. Subsequently, the roots of *A. sinensis* were gently shaken to remove loosely adhering soil, and then the remaining soil tightly attached to the root surface was carefully brushed off with a sterile brush on a laminar flow bench to obtain rhizosphere soil samples (Zhu et al., 2021). Each rhizosphere soil sample was divided into three parts. One portion was placed in sterile self-sealing bags and stored at -80°C for high-throughput sequencing; one part was stored at 4°C for microbial biomass carbon (MC) and microbial biomass nitrogen (MN); the other part was air-dried at room temperature and passed through a 0.149-mm sieve to assess the soil physicochemical properties, soil nutrients, and soil enzymes. The contents of medicinal components in *A. sinensis* were measured after natural drying. All indicators were determined six replicates.

### Physicochemical properties, soil nutrients, and enzyme activities of rhizosphere soil

Soil pH was measured using a pH meter, and the soil-to-water ratio was 1:2.5. The electrical conductivity (EC) was measured using an electrical conductivity meter (Lei-ci PHS-3C, Shanghai Yidian Scientific Instruments Co., Ltd, Shanghai, China) with a soil-to-water ratio of 1:2.5. Soil water content (SWC) was determined by the gravimetric method. Soil MC and MN were calculated using the fumigation-extraction method, MC by a carbon auto-analyzer (Phoenix 8000, Teledyne Tekmar, USA), and MN by a flow injection analyzer (FIAStar 5000, Foss, Denmark), respectively. Organic matter (OM) content of the rhizosphere soil was determined using the H_2_SO_4_-K_2_Cr_2_O_7_ oxidation method. Total nitrogen (TN) was determined using the Kjeldahl method, and Alkali-hydrolyzed nitrogen (AN) was determined by alkaline hydrolysis distillation method with a fully automatic Kjeldahl nitrogen analyzer (K1100, Hanon Instruments, Jinan, China). Available phosphorus (AP) was determined by the molybdenum-antimony colorimetric method using an eight-cell UV spectrophotometer (TU-1950, Beijing Puxi Technology Co., Ltd, Beijing, China). The available potassium (AK) in the soil was determined using the flame photometric method with atomic absorption spectrophotometry (A3AFG-00, Beijing Puxi Technology Co., Ltd, Beijing, China) (Bao, 2011). Urease (Ur) was measured by the sodium phenol-sodium hypochlorite colorimetric method. Catalase activity (Ca) was determined by the potassium permanganate titration method. Sucrase activity (Sa) was quantified using the 3,5-dinitrosalicylic acid colorimetric method. Acid Phosphatase (Acp) was assessed using phenylene disodium phosphate colorimetry. All soil enzyme indices were calculated by ultraviolet spectrophotometry (TU-1950, Beijing Puxi Technology Co., Ltd, Beijing, China).

### Determination of phenolic volatile oil components in *A. sinensis*

The contents of the five phenolic volatile oils in *A. sinensis* roots were determined using high-performance liquid chromatography (HPLC). The chromatographic conditions were as follows: a MerkRP-C18 column (250.0 mm × 4.6 mm, 5 µm) was used with acetonitrile (B)-1% acetic acid (A) as the mobile phase, under gradient elution. The detection wavelength was 280 nm, column temperature was 30°C, flow rate was 1 mL/min, and injection volume was 20 µL (Luo et al., 2021).

### DNA extraction, PCR amplification, and high-throughput sequencing of rhizosphere soil

The E.Z.N.A.^®^ soil DNA kit (Omega Bio-tek, Norcross, GA, U.S.) was used to extract the total genomic DNA of soil samples according to the manufacturer’s instructions. The quality and concentration of DNA were determined by 1.0% agarose gel electrophoresis and a NanoDrop^®^ ND-2000 spectrophotometer (Thermo Scientific Inc., USA) and samples were stored at -80°C before further use. Using the extracted DNA as a template, the hypervariable region (V3–V4) of the 16S rRNA gene of the bacterial community, the internal transcribed spacer (ITS) region of fungi, the hypervariable region (V4–V5) of the 18S rRNA gene of AM fungi, and the hypervariable region (V4–V5) of the 16S rRNA gene of archaea, were amplified using the primer pairs 338F-806R, ITS1F-ITS2R, AMV4-5NF-AMDGR and 524F10extF-Arch958RmodR, respectively (**Table 1**) (Shehata and Newmaster, 2023; Tian et al., 2022; Van et al., 2014; Liu et al., 2021b).

**Table 1:** The sequence information of primer pairs.

Primer types	Primer pairs	Sequence information
Bacteria	338F	5′-ACTCCTACGGGAGGCAGCAG-3′
	806R	5′-GGACTACHVGGGTWTCTAAT-3′
Fungi	ITS1F	5′-CTTGGTCATTTAGAGGAAGTAA-3′
	ITS2R	5′-GCTGCGTTCTTCATCGATGC-3′
AMF	AMV4-5NF	5′-AAGCTCGTAGTTGAATTTCG-3′
	AMDGR	5′-CCCAACTATCCCTATTAATCAT-3′
Archaea	524F10extF	5′-TGYCAGCCGCCGCGGTAA-3′
	Arch958RmodR	5′-YCCGGCGTTGAVTCCAATT-3′

PCR was performed on an ABI GeneAmp^®^ 9700 PCR thermocycler (Applied Biosystems, CA, USA). The PCR reaction system included 4 μL of 5 × TransStart FastPfu buffer, 2 μL of 2.5 mM dNTPs, 0.8 μL of each upstream (5 µM) and downstream (5 µM) primer, 0.4 μL of TransStart FastPfu DNA polymerase, and 10 ng of template DNA, supplemented to 20 μL with ddH_2_O. The PCR amplification cycling conditions were as follows, initial denaturation at 95°C for 3 min, followed by 29 cycles for bacterial primers, 35 cycles for fungal and archaeal primers, and 25 cycles for AM fungal primers, each consisting of denaturation at 95°C for 30 s, annealing at 55°C for 30 s, and extension at 72°C for 45 s. A final extension step was performed at 72°C for 10 min, and then stored at 4°C. Each sample was amplified three times. Subsequently, the PCR products from the same template were pooled, separated on 2% agarose gels, purified using the Axyprep DNA gel extraction kit (Axygen Biosciences, Union City, CA, USA), and then quantified using a Quantus ™ Fluorometer (Promega, USA). The purified amplification products were combined in equimolar amounts, and paired-end sequencing was performed using a MiSeq PE300 platform (Illumina, San Diego, USA) according to the standard protocols of Majorbio Bio-Pharm Technology Co., Ltd. (Shanghai, China).

### Data processing and statistical analysis

Fastp (https://github.com/OpenGene/fastp, version 0.19.6) was used to control the quality of the raw paired-end reads (Chen et al., 2018). Subsequently, FLASH version 1.2.11 (Magoč and Salzberg et al., 2011) was used to merge overlapping paired-end reads. Following this, UPARSE (http://drive5.com/uparse/, Version 7.1) was used to cluster quality-controlled concatenated sequences into Operational Taxonomic Units (OTUs) based on a similarity threshold of 97%, while eliminating chimeric sequences (Edgar, 2013). To mitigate the impact of sequencing depth on subsequent analyses of Alpha and Beta diversity, the sequence number of all samples was rarefied, and each sample still yielded an average coverage of 97.00%. The Ribosomal Database Project (RDP) classifier (http://github.com/rdpstaff/classifier, version 2.13) was then used for comparison with the Silva 16S rRNA (v138) database for bacterial and archaeal sequences, MaarjAM database for AM fungal sequences, and Unite 9.0 databases for fungal OTU species taxonomy annotation, respectively, with a confidence threshold of 70% to calculate the community composition of each sample at the different taxonomic classification levels (Wang et al., 2007).

Based on the OTU information, alpha (α) diversity indices, including the Shannon index and ACE richness, were calculated using Mothur v1.30.1 (Schloss et al., 2009). To evaluate the beta (β) diversity of microorganisms, we employed non-metric multidimensional scaling analysis (NMDS) based on Bray Curtis dissimilarity matrix, using the Vegan v2.5–3 package. In addition, LEfSe was used to calculate the multilevel species discriminant analysis of microbial communities and to identify taxa with significantly different relative abundances among elevations (Han et al., 2024; Ren et al., 2022). Soil physicochemical properties, nutrients, and medicinal ingredients were analyzed using SPSS 23 software, and Student’s t-test was used for pairwise comparisons, while one-way ANOVA was applied for multi-group comparisons across elevations. Redundancy Analysis (RDA) was used to investigate the correlations between rhizosphere soil microbial communities and soil factors. Moreover, the Mantel test was performed to analyze the correlations between the dominant rhizosphere soil microbial genera, soil factors, and medicinal ingredients (with high content and significant differences, *p* < 0.05). Diversity, composition, and species difference maps of bacteria and fungi were generated using R software. The Mantel test correlation analysis was conducted using ChiPlot software (https://www.chiplot.online/). Nonlinear regression modeling of the effects of elevation on root weight and bioactive component concentrations was performed using Origin 9.0 (Origin Lab, USA). Finally, SEM was adopted to evaluate the effects of elevation on soil characteristics (including soil indicators and soil microbial communities) and medicinal ingredients (which showed significant differences between different cultivation elevations, *p* < 0.05) with the piecewise SEM package.

## Results

### Diversity of microorganisms in the rhizosphere soil of *A. sinensis* at different cultivation elevations

**Figure 1:**
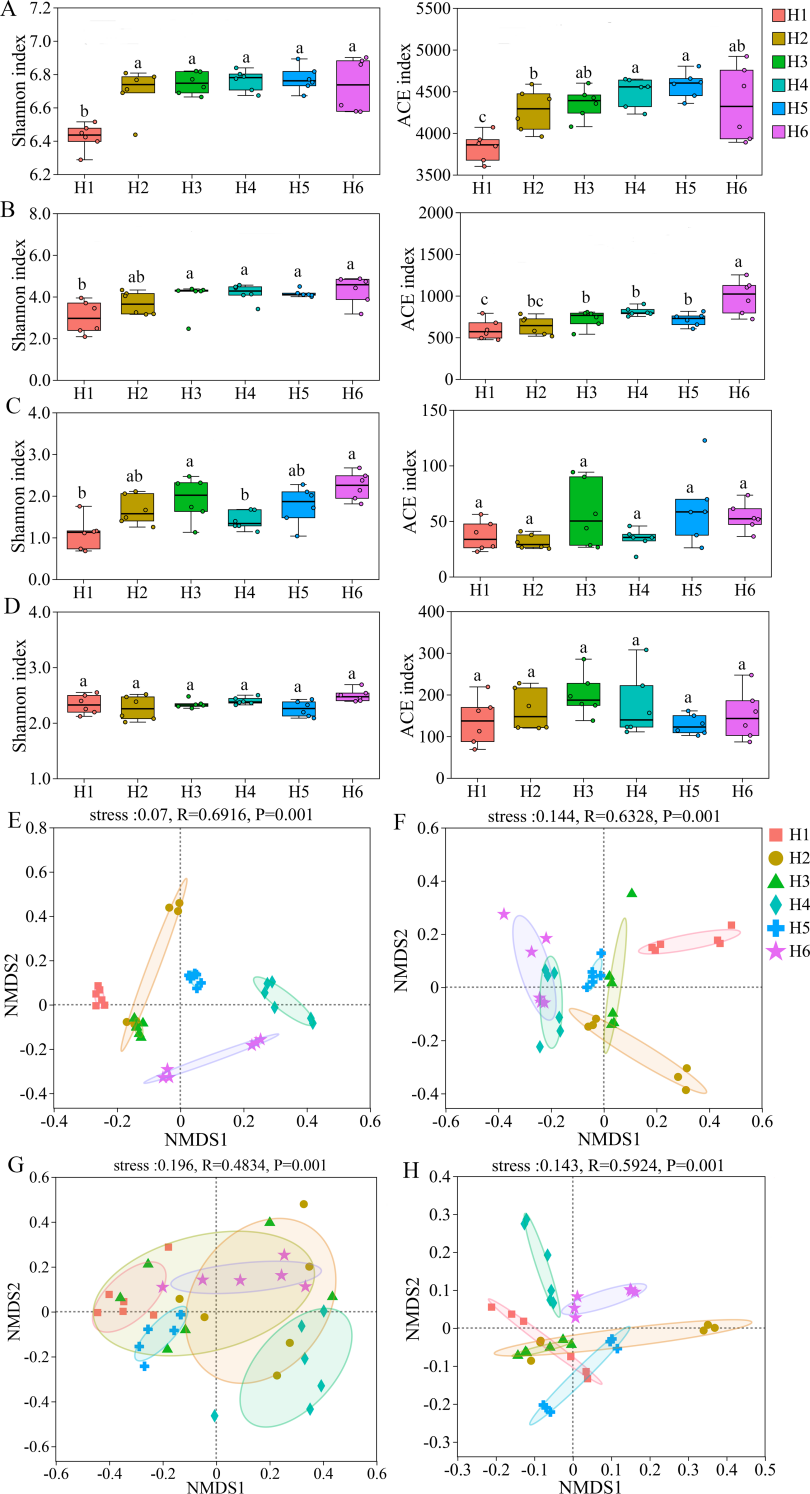
Diversity of microbial communities in the rhizosphere soil of *A. sinensis* at different cultivation elevations. Significant differences among means were evaluated by one-way ANOVA followed by Fisher’s least significant difference (LSD) test (*p* ≤ 0.05; n = 6 per elevation); different lowercase letters indicate statistically significant differences at the at *p* ≤ 0.05; the same letters denote no significant differences. H1: 2210 m, H2: 2425 m, H3: 2520 m, H4: 2615 m, H5: 2717 m, and H6: 2887 m. **(A–D)** α-diversity (Shannon and ACE indices) for bacterial **(A)**, fungal **(B)**, AM fungal **(C)**, and archaeal **(D)** communities. **(E–H)** β-diversity of the corresponding microbial communities based on Bray–Curtis dissimilarities (bacteria: **E**; fungi: **F**; AM fungi: **G**; archaea: **H**).

The α-diversity index measures the diversity and richness of microbial communities. In this study, the Shannon index for bacteria in H1 was significantly lower compared to H2 through H6. Additionally, no significant differences were observed among the indices of H2 to H6. The ACE index showed an upward trend from H1 to H6. Specifically, H2 was significantly lower than H4, H5, and H6, while H1 was significantly lower than H3, H4, H5, and H6. However, no significant differences were found between H1 and H2, H2 and H3, or H3 and H6 (*p* < 0.05, **Figure 1A**). The Shannon index for fungi increased consistently from H1 to H6. H1 was significantly lower than H3 to H6, while no significant differences were found between H2 and H6. The ACE index demonstrated an increase from H1 to H6, with H6 being significantly higher than H1 to H5. Furthermore, the values from H3 to H5 were also higher than those of H1. However, no significant differences were observed between H1 and H2, as well as between H3 and H5 (*p* < 0.05, **Figure 1B**). For AM fungi, the Shannon index showed only limited variation among elevations, with a few pairwise comparisons showing significant differences, whereas no significant differences were detected between H1 and H2, H2 and H3, H4 and H5, or H5 and H6. The ACE index showed no significant differences among elevations (**Figure 1C**). For archaea, no significant differences were observed in either diversity (Shannon) or richness (ACE) indices across the six elevations (**Figure 1D**). These findings indicate that the diversity and richness of bacterial and fungal communities in the rhizosphere soil of *A. sinensis* show an overall increasing trend with elevation, whereas AM fungal and archaeal communities remain relatively stable. Thus, the response of α-diversity and richness to elevation is more pronounced for bacteria and fungi than for AM fungi and archaea.

The β-diversity of bacterial, fungal, AM fungal, and archaeal communities in the rhizosphere soil of *A. sinensis* was analyzed at different cultivation elevations using Bray–Curtis dissimilarities and NMDS, with ANOSIM employed to test for differences among elevations. The NMDS results showed that, except for AM fungi, the grouping ellipses of H1–H6 were clearly separated in the ordination space for bacteria, fungi, and archaea (**Figures 1E–H**). All NMDS analyses had stress values < 0.2, and ANOSIM indicated significant differences in community structure among elevations for bacteria (R = 0.6916, *p* = 0.001), fungi (R = 0.6328, *p* = 0.001), and archaea (R = 0.5924, *p* = 0.001). These findings reveal notable elevational differences in bacterial community structure, with more moderate but still detectable variations in fungal and archaeal communities, while AM fungal communities showed only minor differences among elevations.

### Microbial composition analysis in the rhizosphere soil of *A. sinensis* at different cultivation elevations

**Figure 2:**
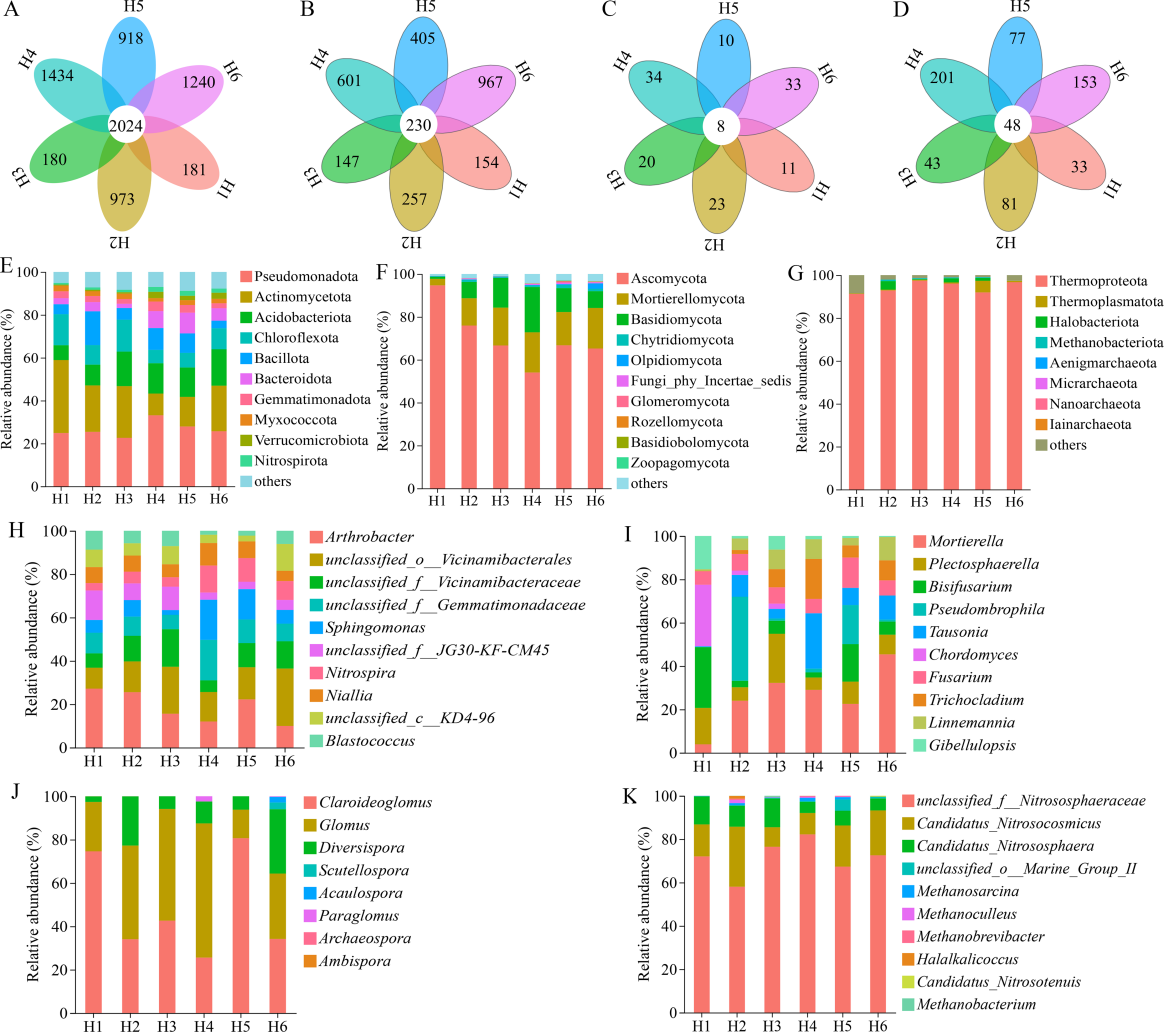
Composition of bacteria, fungi, AM fungi and archaea in the rhizosphere soil of *A*. *sinensis* at different cultivation elevations. H1: 2210 m, H2: 2425 m, H3: 2520 m, H4: 2615 m, H5: 2717 m, and H6: 2887 m; n = 6 per elevation; **(A–D)** Venn diagrams of OTU of bacteria **(A)**, fungi **(B)**, AM fungi **(C)** and archaea **(D)**; **(E–G)** stacked bar plots showing the relative abundance of dominant phyla of bacteria **(E)**, fungi **(F)**,and archaea **(G)**; **(H–K)** show the relative abundance of the top 10 most abundant genera of bacteria **(H)**, fungi **(I)**, AM fungi **(J)** and archaea **(K)** in each group, and the sum of relative abundances for these selected genera equals 100% per sample, and other low-abundance genera are not displayed.

The rhizosphere microbial composition of *A. sinensis* cultivated at different elevations was analyzed (**Figure 2**). The numbers of common and unique OTUs for bacteria, fungi, AM fungi, and archaea are shown in **Figures 2A-D**, respectively. The number of OTUs showed a general trend of initially increasing and then decreasing from H1 to H6. Except for fungi, the other three types of microbes exhibited their highest OTU numbers at H4. The composition of microbial communities at the phylum level across various cultivation elevations is shown in **Figures 2E-G** and **Table 2**. The dominant bacteria in the rhizosphere soil were similar across different elevations, including Pseudomonadota, Actinomycetota, Acidobacteriota, Chloroflexota, Bacillota, and Bacteroidota. The dominant fungi consisted of Ascomycota, Mortirellomycota, Basidiomycota, Chytridiomycota, and Olpidiomycota. Ascomycota had the highest relative abundance at all elevations (54%), with H1 showing the highest proportion (95.5%). AM fungal communities were composed almost entirely of Glomeromycota at all elevations (H1–H6). The dominant archaea comprised Thermoprotcota. The relative abundance of Thermoprotcota was the highest in H1-H6, with all exceeding 91%, and H3 has the highest proportion, at 98.3%. In addition, the dominant genera (top 10 in relative abundance) microbes in the rhizosphere soil of *A. sinensis* at different cultivation elevations is shown in **Figure 2H-K, respectively**. The dominant bacteria consisted of *Arthrobacter*, unclassified_*Vicinamibacterales*, *Sphingomonas*, unclassified_*Vicinamibacteraceae*, unclassified_*Gemmatimonadaceae*, *Nitrospira*, *Niallia*, and *Blastococcus*. The dominant fungi included *Mortierella*, *Plectosphaerella*, *Bisifusarium*, *Pseudombrophila*, *Tausonia*, *Chordomyces*, *Fusarium, Trichocladium, Linnemannia*, and *Gibellulopsis.* Compared to H3-H6, there were apparent differences in the compositional abundance of H1 and H2 especially for several dominant fungal genera. The dominant AM fungi mainly consisted of *Claroideoglomus*, *Glomus*, and *Diversispora*, and there were larger differences in the compositional abundance of H1 and H5 compared to other groups. Besides, the dominant archaea included unclassified*_Nitrososphaeraceae, Candidatus_Nitrosocosmicus*, and *Candidatus_Nitrososphaera*, and there were also noticeable differences in the abundance of different genera among the six groups. These results reveal that the composition of *A. sinensis* rhizosphere soil microbes varied significantly with elevation.

**Table 2:** The differences of microbial species on phylum level in rhizosphere of *A. sinensis* at different cultivated elevations.

Species type	Phylum name	H1 means±SD (%)	H2 means±SD (%)	H3 means±SD (%)	H4 means±SD (%)	H5 means±SD (%)	H6 means±SD (%)
Bacteria	Pseudomonadota	24.89±4.06bc	25.43±1.62bc	22.64±4.86c	33.15±5.53a	27.93±1.86b	25.71±2.12bc
	Actinomycctota	34.02±0.62a	21.65±1.41c	24.11±2.38b	10.13±0.66d	13.81±0.92d	21.25±0.67c
	Acidobacteriota	6.92±1.44c	9.69±0.34b	16.11±5.50a	14.15±4.72a	13.71±2.79a	16.97±0.88a
	Bacillota	4.66±0.59c	15.77±0.97a	5.46±1.29c	10.19±1.11b	9.07±2.34b	3.55±1.04d
	Chloroflexota	14.47±1.06a	9.11±0.55b	14.87±1.42a	6.23±1.15c	6.83±0.48c	9.81±0.82b
	Bacteroidota	2.82±0.16d	4.29±1.17c	2.01±0.75d	7.88±1.22b	9.68±1.41a	5.79±1.07c
Fungus	Ascomycota	94.70±1.33a	75.96±3.87b	66.66±7.40c	54.06±8.24d	66.75±2.14c	65.25±1.11c
	Mortierellomycota	3.03±0.46c	12.73±3.18b	17.66±6.27a	18.75±8.63a	15.51±2.22a	18.91±1.02a
	Basidiomycota	0.93±0.04d	7.61±0.29c	13.93±1.25b	21.12±8.91a	11.15±3.78b	7.82±0.36c
AM fungi	Glomeromycota	100.00±0.00a	100.00±0.00a	100.00±0.00a	100.00±0.00a	100.00±0.00a	100.00±0.00a
Archaea	Thermoproteota	91.26±0.22b	92.84±0.37b	97.42±1.63a	96.06±3.21ab	91.94±4.99b	96.70±0.48a

The date means ± SE, n = 6 per elevation, different lowercase letters in the same line indicate significant differences at 0.05 level, while the same lowercase letters indicate no significant differences at 0.05 level.

### Differences in microbial composition at the species level of *A. sinensis* rhizosphere soil at different cultivation elevations

**Figure 3:**
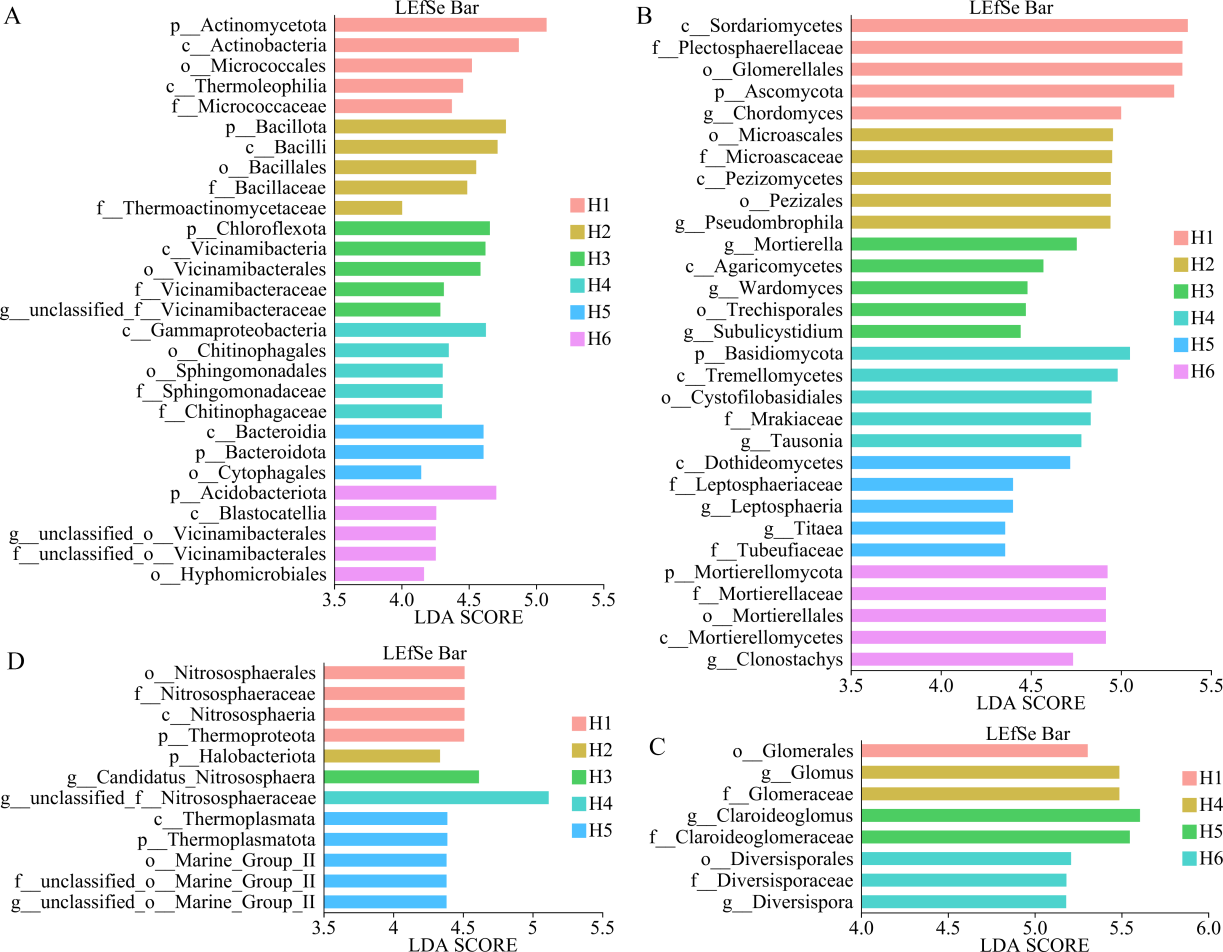
LEfSe analysis of rhizosphere soil microorganisms of *A. sinensis* at different cultivated elevations. LDA > 4. H1: 2210 m, H2: 2425 m, H3: 2520 m, H4:2615 m, H5: 2717 m, and H6: 2887 m; n = 6 per elevation; **(A)** bacteria, **(B)** fungi, **(C)** AM fungi, and **(D)** archaea.

LEfSe analysis was applied to the taxonomic compositions of rhizosphere soil microbial communities to identify differences in species from phylum to genus for *A. sinensis* at different cultivation elevations. A LDA score above 4.0 was selected as the threshold for differential analysis (**Figure 3**). There were more species of bacteria significantly (*p* < 0.05) enriched in H1-H6 from phylum to genus, with the phylum of Actinomycctota enriched in H1. The phylum enriched in H2 was Bacillota. In H3, Choroflexota was an enriched phylum, belonging to the genus unclassified_*Vicinamibacteraceae*. No significant phylum- or genus-level bacterial biomarkers were detected at H4. The phylum enriched in H5 was Bacteroidota. The phylum enriched in H6 was Acidobacteriota, and unclassified*_Vicinamibacterales* at the genus level (**Figure 3A**). Similarly, fungal community composition also exhibited significant elevational niche partitioning (LDA = 4.0, LEfSe), with distinct phylum-genus associations. The phylum Ascomycota dominated in H1, harboring the discriminative genus of *Chordomyces*. There was no phylum enriched in H2, H3, and H5, but the enriched genus in H2 was *Pseudombrophila*, in H3 was *Mortierella*, *Wardomyces*, and *Subulicystidiu*m, while *Titaea* and *Leptosphaeria* were enriched in H5. The phylum Basidiomycota was enriched at H4, with the characteristic genus *Tausonia*. The phylum enriched in H6 was Mortierellomycota, and the genus enriched was *Clonostachys* (**Figure 3B**). However, there were fewer species of AM fungi enriched from phylum to genus, and only H4–H6 showed enrichment at the family and genus levels. H4 was enriched in the genus *Glomus*, H5 in *Claroideoglomus*, and H6 in *Diversispora*, indicating that AM fungi have fewer variations among different groups (**Figure 3C**). Additionally, the group of archaea (H1-H6) from phylum to genus was less enriched than bacteria and fungi, but more than AM fungi. At H1, Thermoproteota was exclusively enriched at the phylum level, with no genus-level specialization detected. At H2, Halobacteriota was enriched at the phylum level, again with no enriched genus. In H3 and H4, *Candidatus Nitrososphaera* and unclassified_*Nitrososphaeraceae* were enriched at the genus level, respectively. At H5, Thermoplasmatota (phylum) and the genus unclassified_Marine_Group_II were enriched (**Figure 3D**). Accordingly, there were significant differences in the enriched groups of bacteria, fungi, and archaea, whereas AM fungi showed comparatively limited enrichment in the rhizosphere microbiota of *A. sinensis* cultivated at different elevations. This indicates that the microbial community structure in the rhizosphere of *A. sinensis* is notably influenced by the altitude at which it is grown.

### Soil physicochemical properties, nutrients, and enzyme activities in the rhizosphere soil of *A. sinensis* at different cultivation elevations

Significant elevational variations (*p* < 0.05) were observed in rhizosphere soil properties of *A. sinensis*, including physicochemical parameters, nutrient availability, and enzyme activities (**Table 3**). Despite minimal latitudinal (LA) variation among sampling sites (range: 34.23°N-37.02°N), marked elevational differentiation emerged. Specifically, H2 showed significantly higher values compared to the other elevation groups (H1, H3-H6), while no significant differences were observed among the remaining sites. Consistent with the altitudinal pattern, the soil pH at H2 was significantly higher than that of other groups. Notably, unlike the generally uniform pH distribution typically observed along elevation gradients, H4 and H6 showed significantly lower values than all other sites (*p* < 0.05). However, no significant difference was detected between H4 and H6, or among the remaining groups (H1, H3, and H5), respectively. The soil EC varied across the altitudinal gradients, with H1 showing the highest value, while H4 and H5 exhibited the lowest. No significant differences were observed between H4 and H5 or among the remaining groups (H2, H3, and H6), respectively. SWC, OM, TN, and AN were all significantly higher in H6 than in other groups (*p* < 0.05), and there was marked inter-group heterogeneity. AP in H2 was significantly higher than in other groups, and there were significant differences between the groups (*p* < 0.05). The content of AK of H5 and H6 was significantly higher than that of H4, and H4 was significantly higher than that of the other groups (*p* < 0.05). The remaining groups did not differ substantially. The soil MN of H6 was significantly higher than others, H2 being significantly higher than H1 and H3-H5, and no significant difference was observed in H3 and H5 (*p* < 0.05). MC content of H2 and H4 was highest, followed by H5, which was significantly higher than the remaining groups (*p* < 0.05), with minimal variation among the remaining groups. Similarly, the soil Ur of H2 was significantly higher than that of other groups (*p* < 0.05), followed by H5, H4, and H6, which were significantly higher than H1 and H3. However, no significant difference was found between H4 and H6, or between H1 and H3. While the soil Ca of only H3 was significantly lower than that of other groups (*p* < 0.05), H1, H2, and H6 were significantly higher than that of H4 and H5, and there were no statistical differences in the three groups (H1, H2, and H6) or the other two groups (H4 and H5). Moreover, H6 outperformed all groups of Sa and Acp, and there were significant differences between other groups (*p* < 0.05).

**Table 3:** Physicochemical properties, nutrient contents and enzyme activities of rhizosphere soil of *A. sinensis* at different cultivation elevations.

Soil indicators	Samples					
	H1	H2	H3	H4	H5	H6
EL/m	2213.33±5.16f	2425±4.47e	2513.33±5.16d	2615±8.37c	2716.67±8.16b	2888±5.61a
LA/°	34.23±0.05c	37.02±0.04a	34.38±0.14c	34.66±0.41bc	35.21±0.62b	34.71±0.03bc
pH	8.23±0.01b	8.56±0.03a	8.06±0.01b	7.55±0.34c	8.18±0.06b	7.42±0.02c
EC/ms·cm^-1^	0.51±0.01a	0.21±0.06d	0.43±0.02b	0.15±0.03e	0.15±0.01e	0.32±0.04c
SWC/%	17.92±0.36d	19.35±0.41c	19.08±0.67c	20.43±0.38b	12.82±0.98e	38.18±0.08a
OM/g·kg^-1^	41.24±1.26b	27.52±1.09c	32.30±1.93bc	40.23±1.26b	26.72±1.65c	59.14±1.19a
TN/g·kg^-1^	2.11±0.07c	2.51±0.11b	1.31±0.10d	2.13±0.23c	1.20±0.16d	5.54±0.22a
AN/mg·kg^-1^	69.12±1.97e	87.57±3.32d	217.67±2.67b	137.69±9.29c	71.24±9.59e	350.13±9.41a
AP/mg·kg^-1^	41.32±0.19b	90.88±0.99a	37.60±0.75b	55.37±3.31b	44.25±5.86b	44.29±0.31b
AK/mg·kg^-1^	181.58±6.48c	224.22±1.88c	286.27±8.91c	324.44±2.58b	816.03±8.61a	803.13±2.65a
MN/mg·kg^-1^	11.35±0.06c	13.60±0.04b	8.00±0.08e	9.68±1.85d	7.94±1.22e	14.76±0.12a
MC/mg·kg^-1^	83.38±0.70d	156.05±0.70a	110.19±3.65c	161.22±5.84a	148.75±5.38b	123.74±3.29c
Ur/U.g^-1^	329.06±11.59d	2551.13±24.70a	359.55±35.70d	1440.32±79.96c	1816.31±16.74b	1477.74±1.21c
Ca/mL·g^-1^	0.82±0.05a	0.86±0.01a	0.48±0.02c	0.70±0.02b	0.71±±0.04b	0.84±0.01a
Sa/U.g^-1^	15.31±0.47e	50.29±0.55c	34.88±0.16d	57.60±0.40b	42.48±0.16d	79.38±0.50a
Acp/U.kg^-1^	30.09±0.73bc	22.45±0.21c	37.16±0.65b	28.83±1.28bc	13.64±0.41d	52.69±0.10a

The date means ± SE, n = 6 per elevation; different lowercase letters in the same line indicate significant differences at 0.05 level, while the same lowercase letters indicate no significant differences at 0.05 level.

### Root weight and content of active ingredients of *A. sinensis* at different cultivation elevations

Significant elevational variations were observed in the root weight (RW) and concentrations of pharmacologically active constituents (*p* < 0.05), with distinct compound-specific distribution patterns (**Table 4**). Notably, RW exhibited a unimodal distribution pattern across cultivation elevations, peaking significantly at H4 (*p* < 0.05), with progressive increases from H1 to H4 followed by declines from H4 to H6. In addition, although ferulic acid (FA) showed no significant differences across elevations, the other medicinal components of *A. sinensis* varied significantly with cultivation elevation. Their concentrations were comparable among H4–H6 and were significantly higher than those in H1–H3 (*p* < 0.05), with no significant differences within H4–H6 or within H1–H3. Studies have shown that CF, a phenolic component with low stability, can easily convert into FA and coniferyl alcohol in *A. sinensis*. It can also undergo mutual conversion with FA in *A. sinensis*. The total amount of FA, including both the converted FA and that derived from CF, is referred to as “total FA”. Hence, it can be inferred that the total FA content in *A. sinensis* cultivated at higher altitudes is likely to be significantly higher. Consistent with this, CF levels were significantly lower at H1 and H2 compared to H3–H6, while H3 showed significantly higher CF levels than H4–H6 (*p* < 0.05). No significant differences were detected between H1 and H2, or among H4–H6. Similarly, levistolide A (LEA) levels were significantly higher at H5 compared to the other groups. H1 and H2 were significantly lower than H3, H4, and H6, while no significant differences were observed between H1 and H2 or among H3, H4, and H6. This indicates that the formation and accumulation of different pharmacodynamic components of *A. sinensis* responded differently to the cultivation elevation.

**Table 4:** Root weight and content of main effective components of *A. sinensis* at different cultivated elevations.

Root weight and content of active ingredient	Samples					
	H1	H2	H3	H4	H5	H6
Root weight (RW/g)	77.32±6.809d	78.04±2.762d	94.10±2.362c	114.51±2.737a	105.45±2.858b	70.19±5.404e
Ferulic acid(FA/mg ·g^-1^)	0.61±0.109a	0.65±0.0068a	0.64±0.026a	0.63±0.061a	0.66±0.017a	0.64±0.051a
Coniferyl ferulate(CF/mg ·g^-1^)	1.01±0.387b	1.16±0.295b	0.76±0.568b	2.11±0.543a	2.08±0.490a	2.01±0.261a
Ligustilide(LI/mg ·g^-1^)	6.42±1.129c	6.59±1.012c	13.85±0.592a	9.46±0.453b	9.61±0.561b	11.97±1.392 b
Levistolide A(LEA/mg ·g^-1^)	0.03±0.0007c	0.03±0.0004c	0.04±0.0009b	0.04±0.0009b	0.05±0.0003a	0.04±0.0005b

The date means ± SE, n = 6 per elevation; Different lowercase letters in the same line indicate significant differences in samples at 0.05 level, while the same lowercase letters indicate no significant differences in samples at 0.05 level.

### Interaction between rhizosphere soil microbial communities, soil environmental factors, and pharmacodynamic constituents

**Figure 4:**
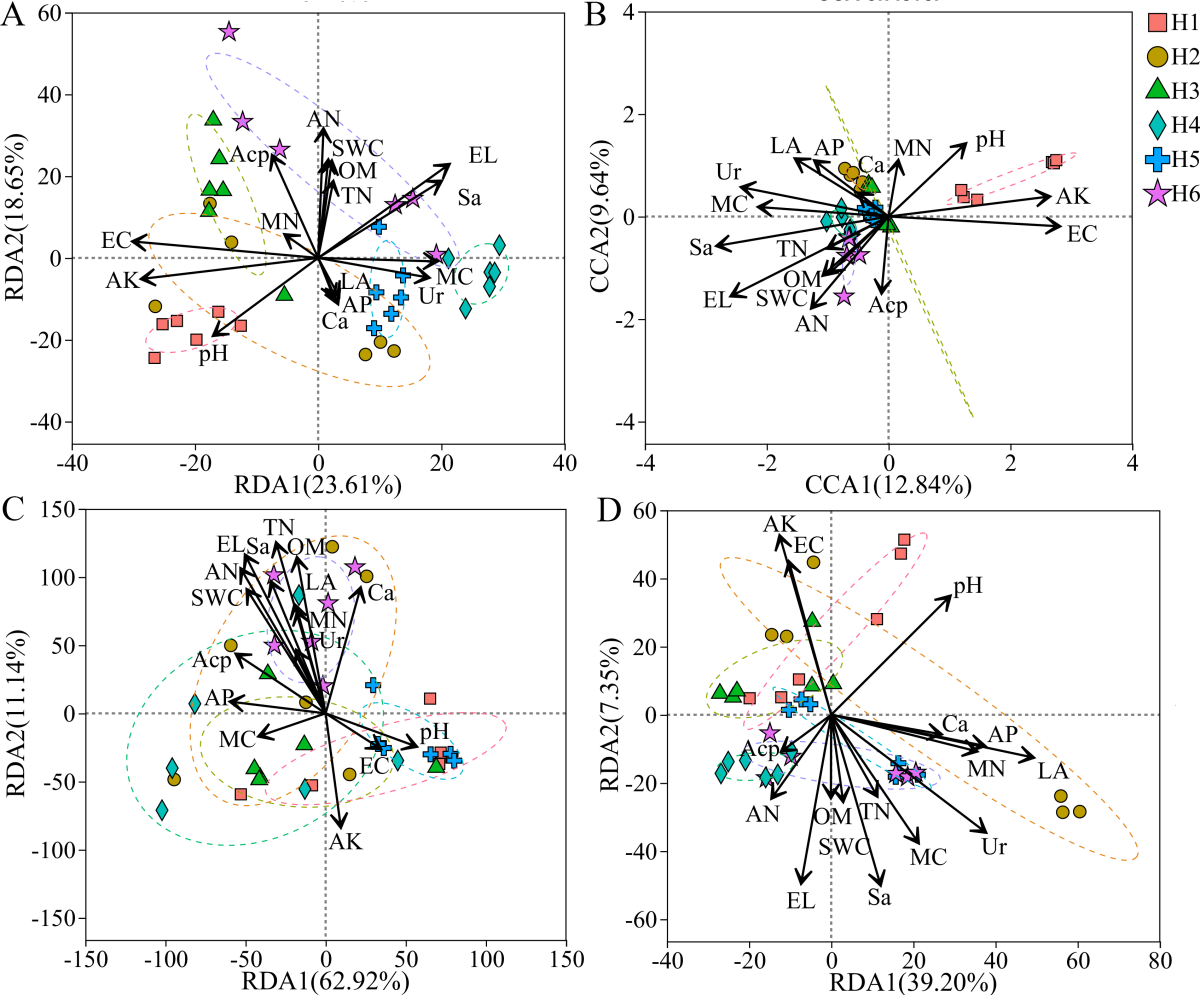
The correlation analysis between soil factors and microbial communities. **(A)** RDA of bacteria; **(B)** CCA of fungi; **(C)** RDA of AM fungi; **(D)** RDA of archaea.

The redundancy analysis (RDA) of microbial community structures and soil environmental factors in the rhizosphere soil of *A. sinensis* was shown in **Figure 4**. The results indicated that RDA1 and RDA2 accounted for 23.61% and 18.65% (a total of 42.26%) of the total variation in bacterial communities, respectively (**Figure 4A**). For fungal communities, CCA1 and CCA2 explained 12.84% and 9.64% (a total of 22.48%) of the total variation, respectively (**Figure 4B**). Furthermore, RDA1 and RDA2 explained 62.92% and 11.14% of the total variation in AM fungal community structure, respectively, collectively accounting for 74.06% of the changes (**Figure 4C**). In contrast, for archaeal communities, the first two axes (RDA1 = 39.20%, RDA2 = 7.35%) collectively explained 46.55% of the changes (**Figure 4D**). These findings support the reliability of the RDA results. As shown in **Figure 4**, soil pH, AP, MC, MN, and Sa showed broadly similar associations with all microbial communities. However, the effects of other soil factors varied across different microbial groups. Specifically, EL had a pronounced impact on bacterial and fungal communities but exerted relatively weaker effects on AM fungi and archaea. LA, EC, and Ur demonstrated stronger influences on fungal, archaeal, and bacterial communities, while their impact on AM fungi was notably weaker. AK had a stronger impact on bacterial and archaeal communities, but exerted less influence on fungal and AM fungal communities. Additionally, AN and Acp significantly affected bacterial, fungal, and AM fungal communities, with a weaker effect observed only on archaea. Conversely, SWC, OM, and TN had substantial impacts on AM fungal communities but exhibited relatively weaker effects on other microbial groups, including bacteria, fungi, and archaea.

**Figure 5:**
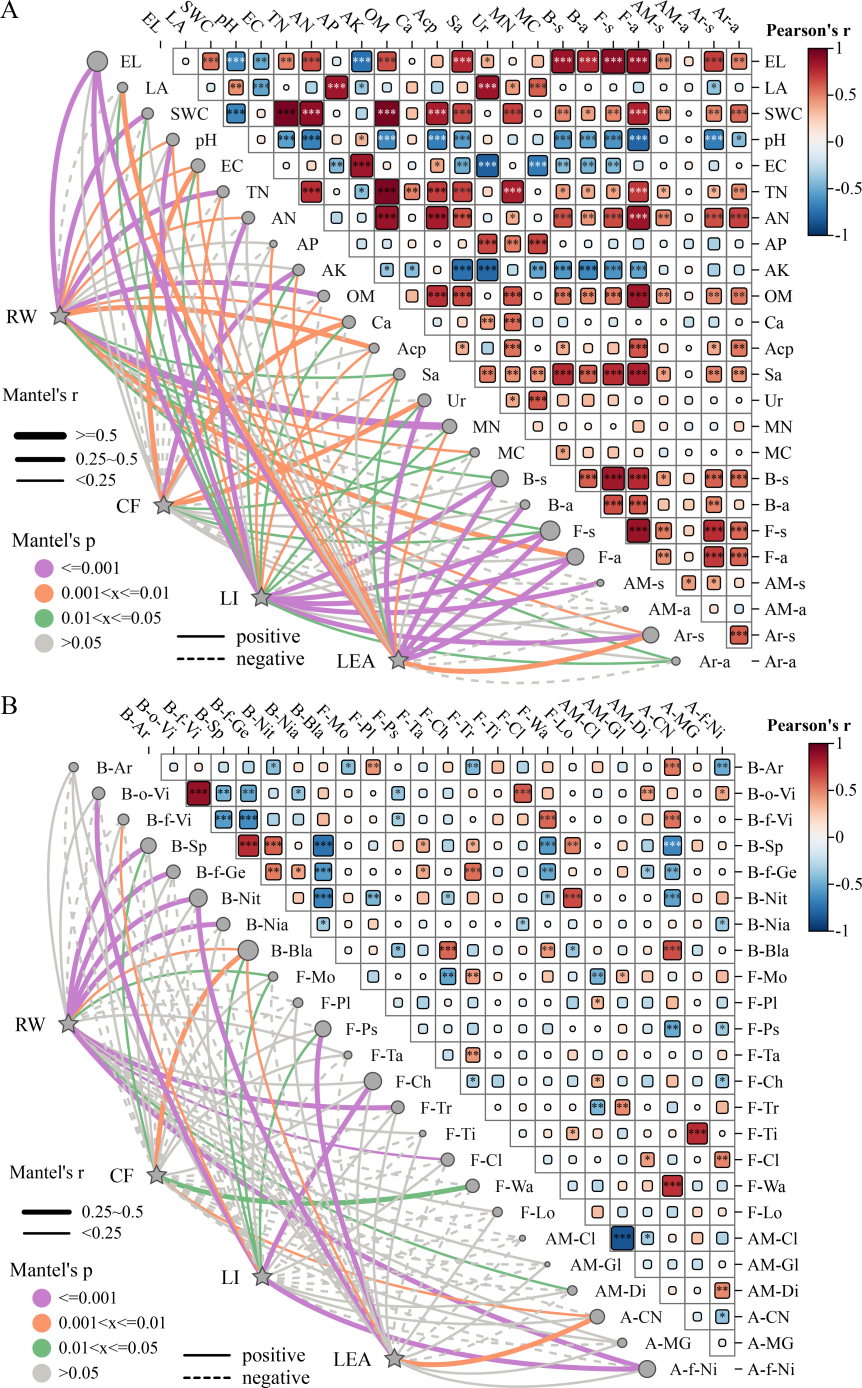
Interaction analysis between soil factors in the rhizosphere soil of *A*. *sinensis* and the content of medicinal components. The Pearson correlation coefficient was calculated in correlation analysis. (*p < 0.05; **p < 0.01; ***p < 0.001). **(A)** Mantel test heatmap of soil factors between pharmacologically active constituents; **(B)** Mantel test heatmap of differential microbial species of dominant genera between pharmacologically active constituents. B-, F-, AM- and Arc-: bacteria, fungi, AM fungi and archaea, respectively; RW: root weight; CF: coniferyl ferulate; LI: ligustilide; LEA: Levistolide A; B/F/AM/Ar-s: bacterial/fungal/AM fungal/archaeal Shannon; B/F/AM/Ar-a: bacterial/fungal/AM fungal/archaeal ACE; B-Ar: g_Arthrobacter; B-o-Vi: unclassified_Vicinamibacterales; B-Nit: g_Nitrospira; B-Sp: g_Sphingomonas: B-f-Vi: unclassified_Vicinamibacteraceae; B-Nia: g_Niallia; B-f-Ge: g:unclassified_f:Gemmatimonadaceae; B-Bla: g:Blastococcus; F-Mo: g_Mortierella; F-Ps: g_Pseudombrophila; F-Pl: g_Plectosphaerella; F-Ta: g_Tausonia; F-Ch: g_Chordomyces; F-Tr: g_Trichocladium; F-Ti: g_Titaea; F-Cl: g_Clonostachys; F-Wa: g_Wardomyces; F-Lo: g_Lophotrichus; AM-Gl: g_Glomus; AM-Cl: g_Claroideoglomus; AM-Di: Diversispora; A-CN: g_Candidatus_Nitrosocosmicus; A-f-Ni: unclassified_f_Nitrososphaeraceae; A-MG: g_norank_o_Marine_Group_II.

The Mantel test was used to assess the correlation between microbial diversity indices, soil factors, root weight, and active ingredients of *A. sinensis*. The resulting heatmap was shown in **Figure 5A**. A significant correlation was observed between environmental factors and microbial diversity indices, particularly for bacterial and fungal indices. Specifically, EL exhibited positive correlations with Ur (*p* < 0.05), AM-s, Ar-a (*p* < 0.01), SWC, TN, AN, OM, Ar-s, Sa, as well as bacterial and fungal diversity indices (*p* < 0.001), while showing negative correlations with EC (*p* < 0.01), pH and AK (*p* < 0.001). LA demonstrated positive correlations with MN (*p* < 0.05), pH (*p* < 0.01), AP, Ur, and MC (*p* < 0.001), and negative correlations with AK, Ar-s (*p* < 0.05), and EC (*p* < 0.001). SWC exhibited a significantly negative correlation with pH (*p* < 0.01), while demonstrating strong positive associations with all other measured soil indices. Notably, SWC showed the most pronounced positive correlations with TN, AN, OM, Acp, Sa, MN, and F/Ar-a (*p* < 0.001). Additionally, pH, EC, and AK were negatively correlated with most of the soil factors. Notably, pH showed highly significant negative correlations with TN, AN, OM, Acp, Sa, B/F-a, and B/F/Ar-s (*p* < 0.001). EC exhibited highly significant negative correlations with Ur and MC, while being positively correlated with AK (*p* < 0.001); AK revealed highly significant negative correlations with Sa, Ur, B/F-s, and B/F-a (*p* < 0.001). Furthermore, TN, AN, OM, Acp, and Sa demonstrated positive correlations with most soil factors. In particular, TN was highly significantly positively correlated with AN, OM, Acp, Sa, MN, and F-a (*p* < 0.001); AN showed highly significant positive correlations with OM, Acp, Sa, B/F/Ar-s, and F/Ar-a (*p* < 0.001); OM exhibited highly significant positive correlations with Acp, Sa, MN, B/F-s, and F-a (*p* < 0.001); Acp showed highly significant positive correlations with MN and F-a (*p* < 0.001); Sa revealed highly significant positive correlations both with B/F-s and F-a indexes (*p* < 0.001). Ur was significantly positively correlated with MC (*p* < 0.001). Lastly, B-s and B-a showed significant positive correlation with F-s and F-a, and Ar-s and Ar-a (*p* < 0.001), while the correlation between AM-s and AM-a was mainly not significant (*p* < 0.05). Moreover, the correlation analysis results between the root weight and pharmacodynamic components of *A. sinensis* and environmental, soil factors, as well as microbial diversity indices revealed that RW, CF, LI, and LEA exhibited significant correlations with these factors. RW exhibited highly significant positive correlations with EL, SWC, TN, OM, MN (*p* < 0.001), pH, EC, AN, Ca, Acp, MC, F-a, Ar-s (*p* < 0.01), Sa, B-s, F-s, and Ar-a (*p* < 0.05). Among the pharmacodynamic components, CF demonstrated highly significant positive correlations with AK (*p* < 0.001), EC, Ca, Ur, Sa (*p* < 0.01), MC, and F-s (*p* < 0.05). LI was significantly positively correlated with EL, pH, AN, B-s, F-s, F-a, AM-s, Ar-s (*p* < 0.001), TN, AK, Acp, Sa (*p* < 0.01), LA, SWC, EC, AK, Ca, Ur, MN, and Ar-a (*p* < 0.05). LEA showed a significantly positive correlation with EL, Ur, B-s, B-a, F-s, F-a (*p* < 0.001), LA, pH, EC, AP, AK, MN, Ar-s (*p* < 0.01), and Sa (*p* < 0.05).

The Mantel test was also used to analyze the correlation between the dominant differential genus of microbes and the characteristic indicators of *A. sinensis* (**Figure 5B**). The results showed a significant correlation between the dominant differential genus of microbes. Specifically, B-Ar in bacteria exhibited a significantly positive correlation with F-P l (*p* < 0.01), and A-CN in archaea (*p* < 0.001), but showed significant negative correlations with B-Nit, F-Mo (*p* < 0.05), F-Tr, and A-f-Ni (*p* < 0.001). B-o-Vi showed significant positive correlations with A-f-Ni (*p* < 0.05), AM-Di (*p* < 0.01), B-f-Vi, and F-Cl (*p* < 0.001), while showing significant negative correlations with B-Nia and F-Ps (*p* < 0.05), as well as B-Sp and B-f-Ge (*p* < 0.01). B-f-Vi was significantly positive correlation with F-Wa, and A-CN (*p* < 0.001), but was significantly negatively correlated with F-Ps (*p* < 0.05), B-Sp, B-f-Ge (*p* < 0.001). B-Sp showed significant positive correlations with F-Ta and F-Tr (*p* < 0.05), F-Lo (*p* < 0.01), as well as B-f-Ge and B-Nit (*p* < 0.001), while showing significant negative correlations with B-Bla, F-Wa, and A-CN (*p* < 0.001). B-f-Ge also showed a significantly positive correlation with B-Nia, F-Ta (*p* < 0.05), B-Nit (*p* < 0.01), F-Tr (*p* < 0.001), but was significantly negatively correlated with AM-Di (*p* < 0.05), F-Wa, A-CN (*p* < 0.01), B-Bla (*p* < 0.001). B-Nit showed a significantly positive correlation with F-Lo (*p* < 0.001), while was significantly negatively correlated with F-Ch, F-Wa (*p* < 0.05), F-Pl (*p* < 0.01), B-Bla, and A-CN (*p* < 0.001). B-Nia showed significant negative correlations with B-Bla, F-Cl, and A-f-Ni (*p* < 0.05). B-Bla exhibited significant positive correlations with F-Wa (*p* < 0.01), F-Ta, and A-CN (*p* < 0.001). Additionally, F-Mo in fungi demonstrated a significantly positive correlation with AM-Gl (*p* < 0.05) and F-Tr (*p* < 0.001), but was negatively correlated with F-Ch, AM-Cl (*p* < 0.01). There was significant positive correlation between F-Pl and AM-Cl (*p* < 0.05) and F-Ps showed a significantly negatively correlated with A-f-Ni (*p* < 0.05), and A-AN (*p* < 0.01). F-Ta was significantly positively correlated with F-Tr (*p* < 0.01). F-Ch demonstrated a significantly positive correlation with AM-Cl (*p* < 0.05), but was a negatively correlated with F-Tr, and A-f-Ni (*p* < 0.05). F-Tr showed a significant positive correlation with AM-Gl and a significant negative correlation with AM-Cl (*p* < 0.01). F-Ti was significantly positively correlated with F-Lo (*p* < 0.05), and A-MG (*p* < 0.001). F-Cl was significantly positively correlated with AM-Di (*p* < 0.05), and A-f-Ni (*p* < 0.01). There was a significant positive correlation between F-Wa and A-CN (*p* < 0.001). AM-Cl in AM fungi was significantly negatively correlated with AM-Di (*p* < 0.05) and AM-Gl (*p* < 0.001). Furthermore, AM-Di in AM fungi showed a significantly positive correlation with A-f-Ni in archaea (*p* < 0.01). Lastly, A-CN in archaea exhibited a significantly negative correlation with archaeal A-f-Ni (*p* < 0.05). In addition, the correlation between the characteristic indicators of *A. sinensis* and the dominant difference genus of microbes showed that RW, LI and LEA were closely related to the soil dominant difference flora. Specifically, B-Sp, B-f-Ge, B-Nit, B-Nia (*p* < 0.001), and B-Bla (*p* < 0.01) in bacteria, F-Tr, F-Cl (*p* < 0.001) in fungi, AM-Di (*p* < 0.05) in AMF, and A-f-Ni (*p* < 0.001) in archaea, were significantly positively correlated with RW. B-Nit (*p* < 0.001), and B-Bla (*p* < 0.01) in bacteria, F-Ps (*p* < 0.001) in fungi, A-CN (*p* < 0.01) in archaea, were significantly positively correlated with LEA. B-Sp (*p* < 0.05) and B-Bla (*p* < 0.01) in bacteria, and F-Wa (*p* < 0.05) in fungi, as well as A-CN (*p* < 0.01) in archaea, were significantly positively correlated with CF. Additionally, B-o-Vi (*p* < 0.001), B-f-Vi (*p* < 0.01), and B-Bla (*p* < 0.05) in bacteria, F-Mo, F-Ps (*p* < 0.05), F-Ch (*p* < 0.001) in fungi, and A-f-Ni (*p* < 0.001) in archaea were significantly positively correlated with LI.

**Figure 6:**
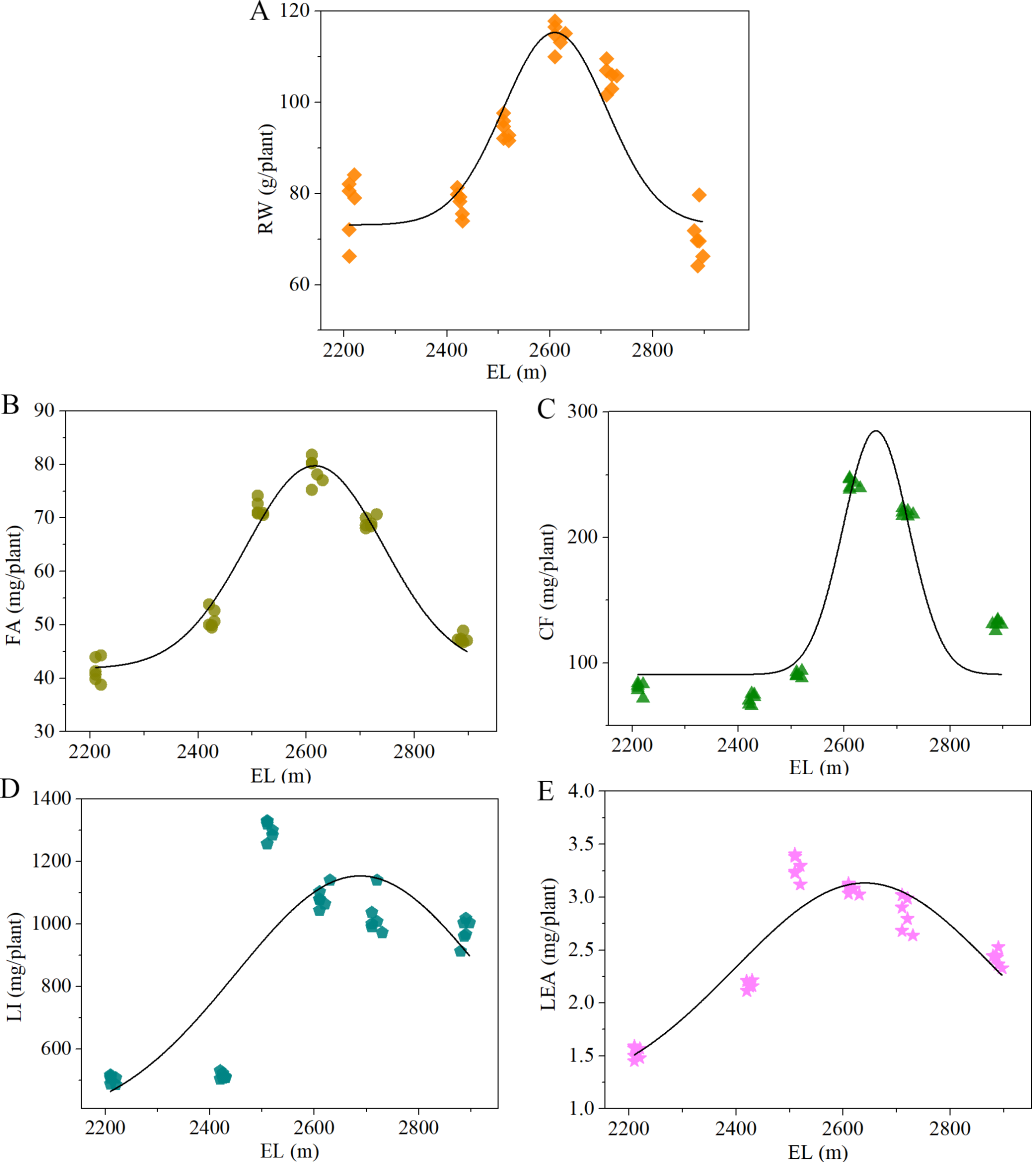
Nonlinear regression modeling for elevation effects on root weight and bioactive compound content of *A. sinensis*. Nonlinear fitting of elevation between root weight **(A),** Ferulic acid **(B)**, Coniferyl ferulate **(C)**, Ligustilide **(D)**, and Levistolide A **(E)**.

Nonlinear regression modeling revealed a Gaussian distribution pattern for elevation-dependent variation of RW (Adj. R² = 0.846) and the bioactive components FA (Adj. R² = 0.963), CF (Adj. R² = 0.897), and LEA (Adj. R² = 0.883) in *A. sinensis*. In contrast, the response of LI (Adj. R² =0.859) to elevation was better described by a Gaussian Amp model (**Figure 6**). All measured parameters exhibited a unimodal trend, initially increasing and subsequently decreasing with increasing elevation, but with distinct elevational optima: RW, FA, and CF peaked at 2615 m, whereas LI and LEA reached their maxima at 2520 m. Notably, the post-peak decline in LI and LEA at higher elevations (> 2615 m) was less pronounced compared to other parameters, indicating that elevated altitudes are more conducive to the production and accumulation of bioactive constituents in *A. sinensis*.

**Figure 7:**
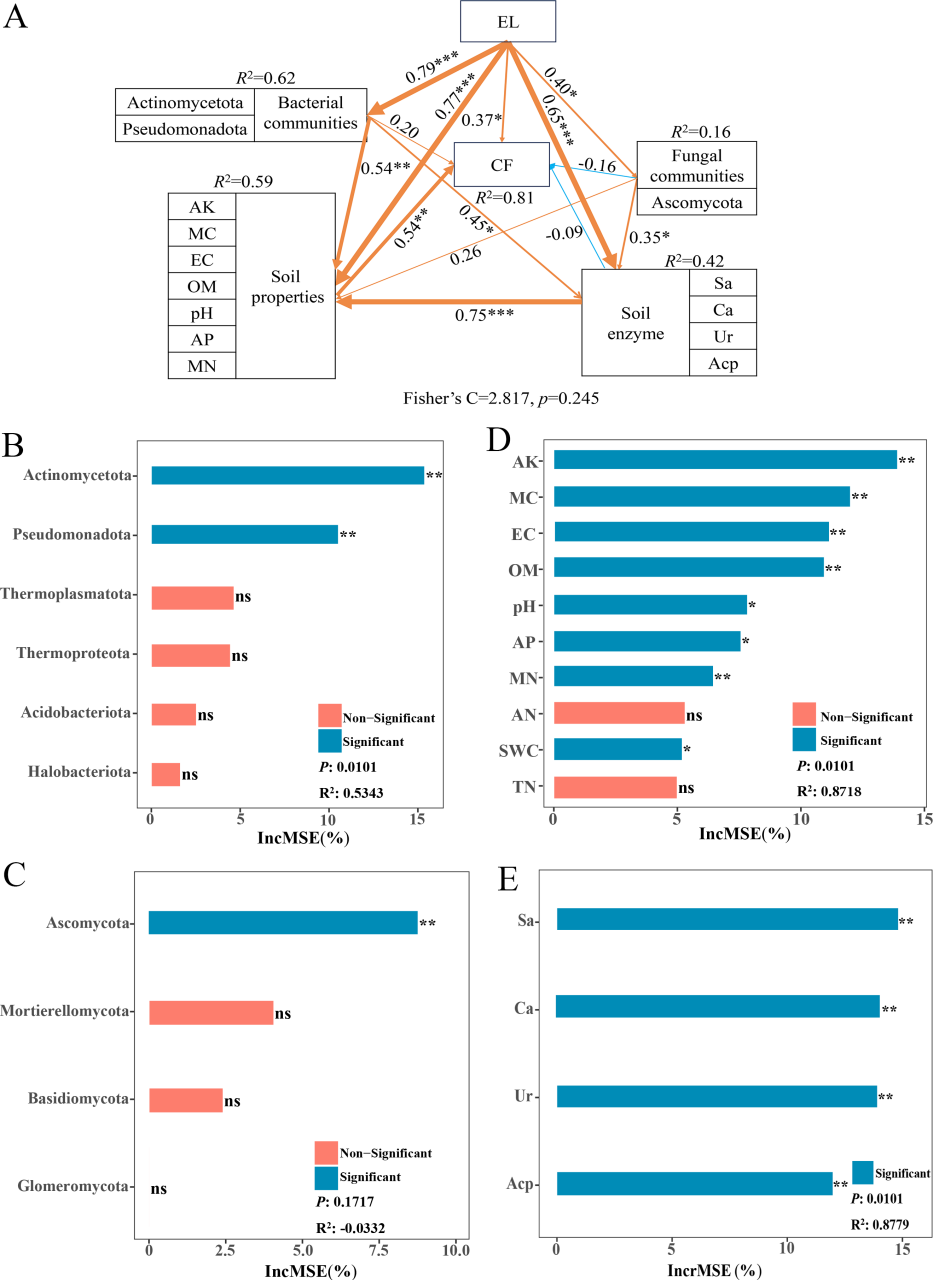
Structural equation models (SEM) showing the direct and indirect effects of elevation, microbial composition, and soil indicators on the content of CF in *A*. *sinensis*. The arrows indicating the hypothesized direction of causality, with the yellow arrows indicating positive effects and blue arrows indicating negative effects. The width of the lines corresponds to the strength of the associations, and numbers adjacent to the lines represent path coefficients. *, ** and *** indicated *p* < 0.05, *p* < 0.01, and *p* < 0.001. The multilayered rectangles indicate the significant predictors of soil indicators and soil microbes. **(A)** SEM of soil factors between CF, predictors importance of influencing factors for content of CF, **(B)** bacterial communities, **(C)** fungal communities, **(D)** soil properties, and **(E)** soil enzyme activities ranked by random forest. Percentage increases in the MSE (mean squared error) of variables were used to estimate the importance of these predictors, and higher MSE% values implied more important predictors.

Moreover, SEM was carried out to determine the main drivers influencing the content of active ingredients of *A. sinensis*. The variables that influence soil microbial communities in the RDA of soil indicators and the dominant phylum composed of microbial communities were chosen as predictors. The SEM analysis further indicated that elevation exerts both direct and indirect effects on the content of active ingredients. The indirect effect is primarily mediated through its influence on the soil microbial community, which subsequently affects the content of medicinal components. Alternatively, elevation may influence soil nutrients and enzyme activity via microorganisms, which in turn affect the content of primary active constituents (**Figure 7**, **S1 and S2**). Dominant phyla Actinomycetota, Thermoplasmatota, and Ascomycota (**Figures 7B, C**, **S1B, S1C, S2B, and S2C**), soil nutrient AK and OM (**Figures 7D**, **S1D, and S2D**), as well as soil enzyme activities (**Figure 7E**), were the most significant and influential factors affecting the content of all active ingredients. Therefore, the content of medicinal ingredients in *A. sinensis* is influenced by the synergistic effects of soil microorganisms, soil nutrients, and soil enzyme activities in growth environments along the elevational gradient.

## Discussion

### Rhizosphere soil microbial properties under varying elevations

Elevation can significantly affect soil microbial diversity, community structure, and functions (Hou et al., 2024; Wei et al., 2024). Our findings demonstrate that elevation gradients induce nonlinear variation patterns in rhizosphere microbial diversity of *A. sinensis* (**Figures 1A-H**), with bacterial and fungal α-diversity exhibiting higher and more stable values at mid- to high elevations (2520–2887 m). This aligns with previous research findings indicating that the rhizosphere of traditional Chinese medicinal plants exhibits variations in microbial diversity along altitudinal gradients (Liu et al., 2024; Pu et al., 2022; Guo et al., 2020), However, it contrasts with other observations describing changes in rhizosphere bacterial and fungal diversity of *Panax ginseng* across different elevations in the Jiuzhai valley forest understory (Jin et al., 2024). This highlights the unique adaptation of *A. sinensis* to high-altitude regions (Xu et al., 2021a). Additionally, the stability of AM fungal and archaeal communities exhibited a negligible response to elevation (**Figures 1C-H**), suggesting their roles as keystone mutualists in buffering *A. sinensis* against altitudinal stressors (Liu et al., 2015; Liu et al., 2023; Xie et al., 2025; Nicol et al., 2005).

Furthermore, the relative abundances of dominant bacterial and fungal taxa responded strongly to elevational variation (**Figure 2**, **Table 2**). The dominant bacteria are likely stable and form a symbiotic community resulting from long-term interaction between *A. sinensis* and soil microorganisms (Mu et al., 2022; Zhu et al., 2021). This community performs a range of physiological functions, including iron chelation, phosphorus solubilization, and potassium mobilization (Karagöz et al., 2012). It contributes to the synergistic absorption of mineral elements by medicinal plants and plays both direct and indirect roles in promoting plant growth as well as the formation and accumulation of bioactive compounds in *A. sinensis* (Xie et al., 2022; Jiang et al., 2024). This, in turn, enabling plant adaptability to the environment (Li et al., 2024b), potentially mediated through root exudates that drive the recruitment of beneficial microbes (Qin et al., 2025).

### Soil properties regulate the composition of microbial communities and bioactive components of *A. sinensis*

In this study, soil pH has a significant effect on the diversity of bacterial, fungal, and archaeal communities in the rhizosphere soil of *A. sinensis* (**Table 3**, **Figures 4**, **5A**). This finding is consistent with previous studies (Jiang et al., 2020; Zhang et al., 2022b; Lauber et al., 2009), indicating that soil pH is a key factor influencing microbial community composition in the rhizosphere. Xu et al. (2021b) demonstrated that soil electrical conductivity is a crucial factor influencing soil microbial diversity and community structure. Higher soil EC can affect the growth and reproduction of microbial communities, reducing microbial abundance (Xu et al., 2021b; Aurélien et al., 2022). However, our results differ showing that microbial diversity and richness indices across different cultivation areas did not increase with decreasing soil EC levels (**Table 3**, **Figures 4**, **5A**). This may be attributed to the fact that the soil conductivity of *A. sinensis* regions cultivated in this study is within the adaptive range of soil bacteria. Consequently, these variations do not impact the sensitivity or activity of the soil bacteria. Therefore, soil conductivity was not the primary factor driving changes in the soil bacterial and fungal communities in this study.

Tan et al. (2023) reported that soil microbial diversity and community composition serve as critical determinants of soil quality and exert a substantial influence on crop growth. Our study found that soil nutrients and enzyme activities, including soil OM, AN, AK, and soil sucrase, were significantly related to soil bacteria and fungi diversity and richness (**Figure 5A**). These results indicate that soil nutrients, microbial diversity, and community functions are interdependent and mutually influential (Yan et al., 2024; Li et al., 2024c). Specifically, soil nutrients provide a favorable environment for microbial metabolism, metabolic activity, and soil enzymes, while microbial activity alters soil chemistry, which in turn shapes microbial community structure (Meng and Qin, 2020; Wang et al., 2020). Furthermore, Shen et al. (2023) showed that variations in soil bacterial diversity may be associated with changes in plant growth and ambient temperatures. Across different elevations originating from the same source area, elevation acts as a direct environmental factor driving temperature variation (Li et al., 2024b). Our findings confirm that elevation is the primary environmental factor responsible for changes in soil properties and other environmental variables, which in turn affect bacterial diversity and the composition of bacterial and fungal communities in the *A. sinensis* rhizosphere (**Figures 5A**, **7A**).

Previous studies have shown that soil provides the essential nutrients for plants (Tong et al., 2021) and its nutritional status greatly influences plant growth (Tsachidou et al., 2019). Soil organic matter is an important source of soil nutrients and an essential component for the growth of traditional Chinese medicinal plants. It plays a significant role in enhancing the accumulation of their bioactive compounds (Zhang et al., 2019; Yu et al., 2024; Wang et al., 2024b). In addition, previous studies have shown that N, P, and K are essential for the growth and production of Chinese medicinal plants (Zhang et al., 2019). In this study, soil properties significantly influence root weight and the contents of coniferyl ferulate, levistolide A, and ligustilide of *A. sinensis*. OM and AK made the most substantial contributions to the soil effect on root weight and the accumulation of these bioactive compounds (**Figures 5A**, **7A**; **Supplementary Figures S1A, S2A**). Additionally, AK was significantly and positively correlated with the contents of ligustilide, coniferyl ferulate, and Levistolide A (**Figure 5A**). It is speculated that root weight and bioactive compound contents are mainly affected by soil properties, with AK and OM being the main regulatory factors for the formation and accumulation of the bioactive compounds in *A. sinensis*.

### Microbial regulation of bioactive components accumulation: agroecological implications for precision cultivation of *A. sinensis*

Rhizosphere microorganisms are crucial for synthesizing the main active natural components of medicinal plants such as *Astragalus*, and some microbes may be developed and utilized as potential medicinal resources (Ding et al., 2024). Furthermore, Sun et al. (2024) demonstrated that a reduction in rhizosphere bacterial diversity led to the inhibition of wogonoside. Consistent with previous research (Ding et al., 2024; Sun et al., 2024), our findings (**Figure 5A**) show that the diversity of microbial flora in the rhizosphere soil of *A. sinensis* plays a crucial role in driving the formation and accumulation of medicinal components in *A. sinensis*. Additionally, *Bacillus* species can produce substances inhibiting plant pathogens and thereby indirectly promote the growth of *A. sinensis* (Rajer et al., 2017). *Pseudomonas* (Cai et al., 2021), *Sphingomonas* (Zhu et al., 2021; Lombardino et al., 2022), *Mortierella* (Yan et al., 2024; He et al., 2025), and *Vicinamibacterales* (Wu et al., 2022) have been proven to promote the growth and accumulation of medicinal compounds in *A. sinensis*. This aligns with our findings (**Figure 5B**) indicating that various rhizosphere bacterial genera significantly and positively correlate with root weight and the content of medicinal components in *A. sinensis*. The mechanism involves soil microbial diversity enhancing nutrient contents and soil enzyme activities, thereby increasing nutrient availability for plants and promoting both plant growth and metabolite accumulation.

Although *Bisifusarium* and *Plectosphaerella* are pathogens of *A. sinensis* (Cheng et al., 2025), their relative abundances are greater at elevations below 2520 m, gradually decreasing as altitude increases above this level. Moreover, Wang et al. (2012) discovered that *A. sinensis* grows most effectively at medium altitudes rather than at low altitudes, likely due to its physiological traits, which are adapted to cooler conditions. Cultivating *A. sinensis* in mid- to high-altitude areas, specifically at elevations of 2520–2887 m, is most beneficial for the functioning of soil microbial communities in the rhizosphere and is the most suitable for the growth and medicinal content of *A. sinensis*. In addition, healthy and vigorously growing plants tend to recruit more dominant bacterial populations by releasing larger amounts of root exudates (Pang et al., 2021). This process enhances their ability to withstand biotic and abiotic stresses and directly or indirectly influences the production of secondary metabolites. Therefore, integrative analysis of soil community structure, nutrient dynamics, and plant growth performance across elevation gradients revealed that middle altitudes (2520–2717 m) are more favorable for *A. sinensis* growth, providing suitable conditions for the formation and accumulation of pharmacodynamic components.

## Conclusion

This study systematically clarified, for the first time, the interaction among soil characteristics, microbial communities, and the bioactive compound content of *A. sinensis* across different altitudes. The study also addresses the limitations of traditional single-factor environmental analyses by providing a more integrated understanding of these complex relationships. The results indicate that soil properties are the main factors influencing the accumulation of bioactive compounds in *A. sinensis*. Among these, soil organic matter (OM) and available potassium (AK) act as key positive regulators of the formation and accumulation of these compounds. Notably, bacterial, fungal, and archaeal communities exhibited significant altitudinal differentiation, whereas AM fungi demonstrated a rare degree of vertical zonal stability. Dominant microbial phyla, including *Pseudomonadota, Actinomycetota, Acidobacteriota, Ascomycota, Mortierelomycota, Glomeromycota*, and *Thermoproteota*, were consistently detected in the rhizosphere of *A. sinensis* across different elevations. Furthermore, the diversity and richness of bacteria, fungi, and archaea in rhizosphere soil were positively correlated with the concentrations of coniferyl ferulate, ligustilide, and levistolide A. By integrating environmental variables and the rhizosphere microenvironment of *A. sinensis*, the mid- to high- elevation transition zone (2520–2717 m) was identified as the optimal cultivation region for maximizing both yield and quality. The altitude–soil–microorganism–plant bioactive compound framework established in this study provides original and valuable insights into enhancing the cultivation and medicinal efficacy of *A. sinensis* across varying altitudes, offering further evidence in support of the theory of microbial symbiosis in high-altitude medicinal plants.

## Data Availability

The datasets presented in this study can be found in online repositories. The names of the repository/repositories and accession number(s) can be found below: https://www.ncbi.nlm.nih.gov/, PRJNA1347083.
