# Supplementary figures and images for: Altitude-mediated soil microbe-nutrient dynamics shape medicinal properties of Angelica sinensis
Source: Front Plant Sci. 2026 Jan 28;16:1703258. doi: 10.3389/fpls.2025.1703258 (PMC12893350; doi:10.3389/fpls.2025.1703258)

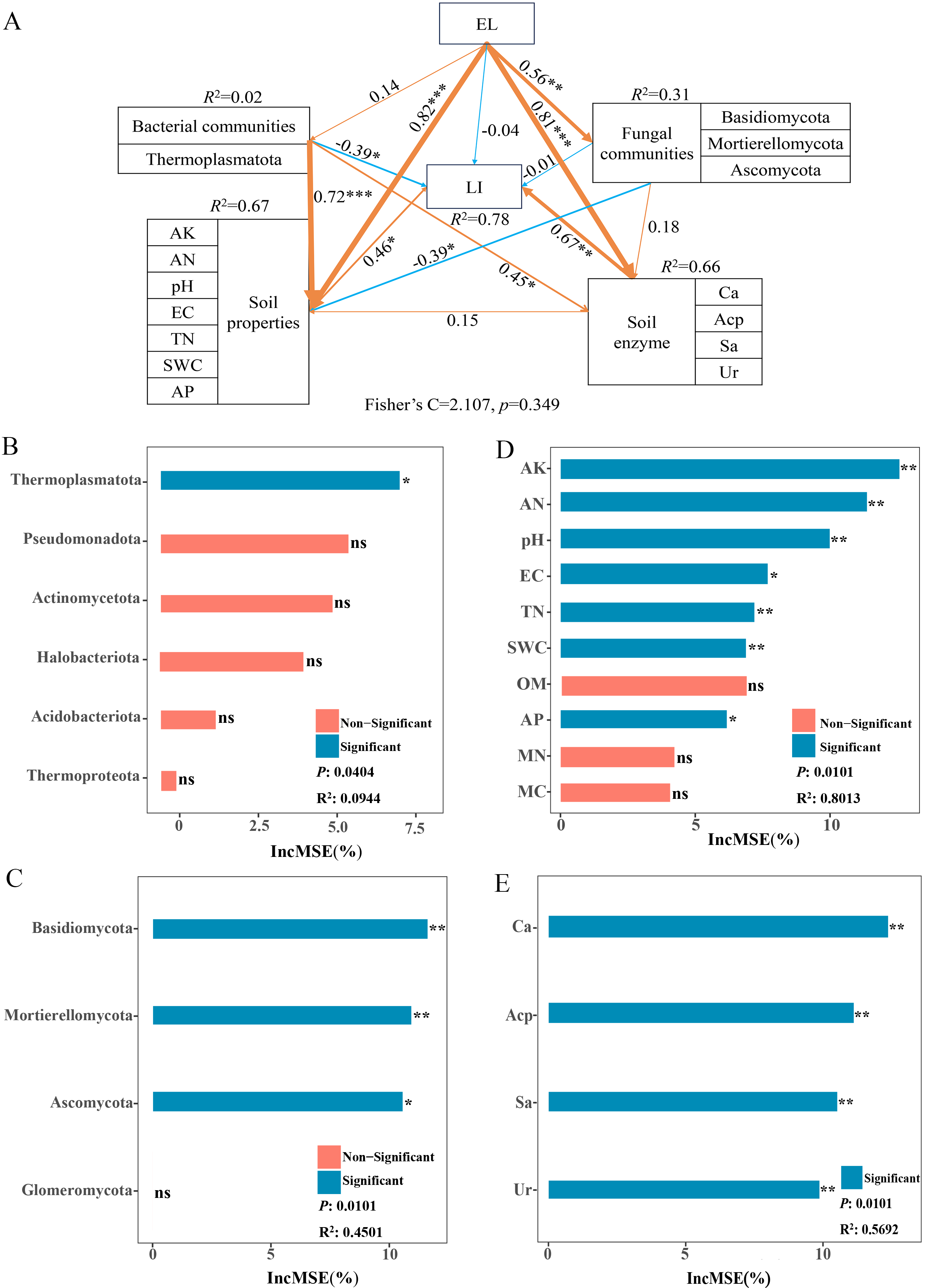

Supplement: Supplementary file 1 [file DataSheet1.zip › Fig.S1.tif]

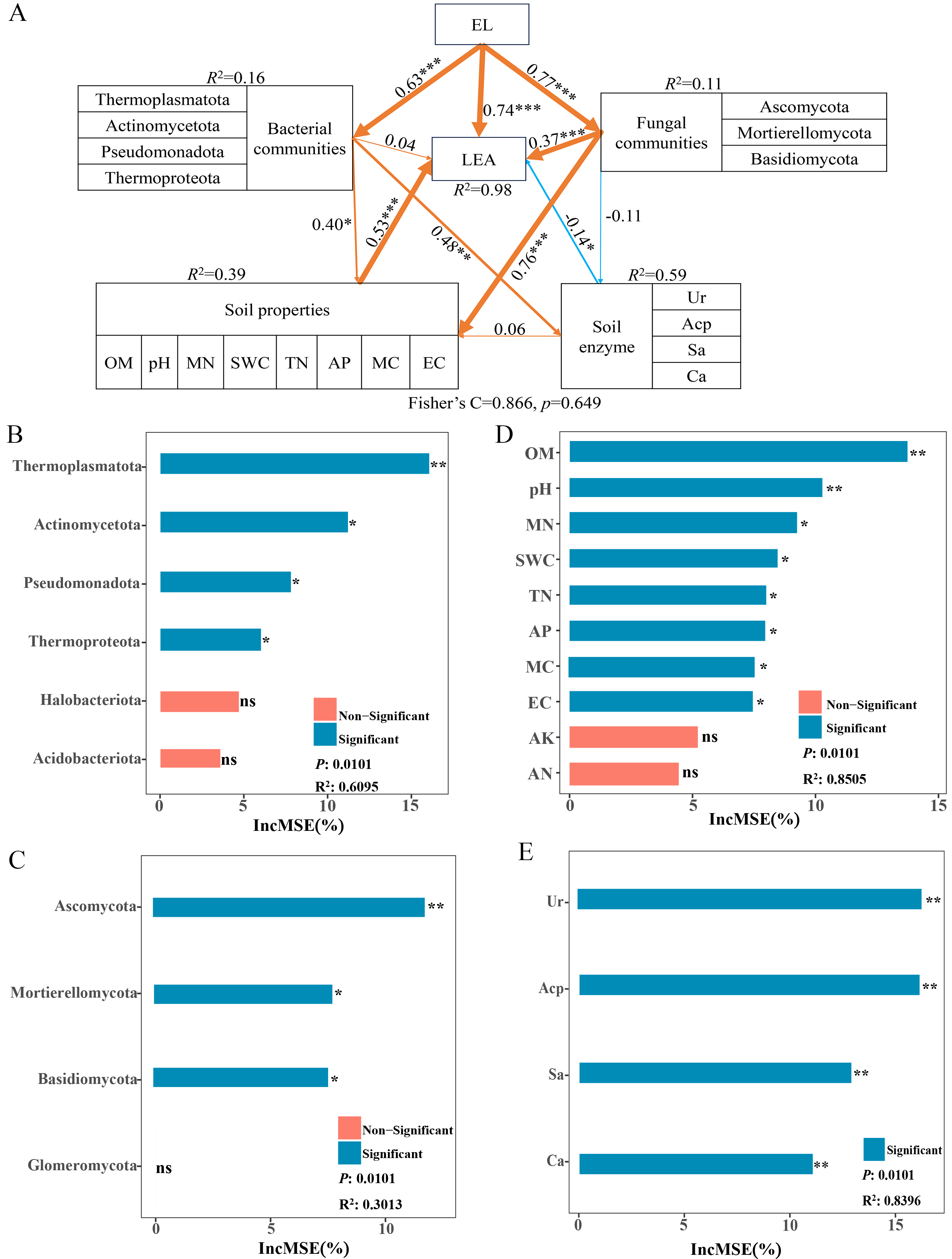

Supplement: Supplementary file 1 [file DataSheet1.zip › Fig.S2.tif]
